# Clinical significance of PD-L1 expression in serum-derived exosomes in NSCLC patients

**DOI:** 10.1186/s12967-019-2101-2

**Published:** 2019-10-29

**Authors:** Chuling Li, Chuwei Li, Chunchun Zhi, Wenjun Liang, Xuan Wang, Xi Chen, Tangfeng Lv, Qin Shen, Yong Song, Dang Lin, Hongbing Liu

**Affiliations:** 1 Department of Respiratory Medicine, Jinling Hospital, Nanjing Medical University, Nanjing, China; 2 Department of Basic Medical Sciences, Chengdu Medical College, Chengdu, China; 3 Department of Pathology, Jinling Hospital, Nanjing University School of Medicine, Nanjing, China; 4 Department of Respiratory Medicine, Affiliated Changzhou Second Hospital of Nanjing Medical University, Changzhou, Jiangsu China; 5 Department of Respiratory Medicine, Jinling Hospital, Nanjing University School of Medicine, Nanjing, China; 6 Department of Respiratory and Critical Care Medicine, Affiliated Suzhou Hospital of Nanjing Medical University, Suzhou, China

**Keywords:** Exosomes, PD-L1, Immunotherapy, NSCLC

## Abstract

**Background:**

Exosomes are 50–150 nm endocytic vesicles secreted by almost all type of cells that carry bioactive molecules from host. These small vesicles are considered to be novel cross-talk circuits established by tumor cells and tumor microenvironment. Previous studies have shown certain biological influence of exosomal programmed cell-death ligand 1 (Exo-PD-L1) on immune suppression and dysfunction. The aim of the current study was to investigate the impact of Exo-PD-L1 and soluble PD-L1 (sPD-L1) on non-small cell lung cancer (NSCLC) and explore the concordance between Exo-PD-L1 and PD-L1 expression in matched tumor tissues in NSCLC patients.

**Methods:**

85 consecutive patients from April 2017 to December 2017 at General Hospital of Eastern Command Theatre who were primarily diagnosed with NSCLC and 27 healthy individuals were enrolled in this study. Two milliliters of whole blood samples were collected from each participant and further centrifuged. Exosomes were derived from serum using the commercial kit (Total Exosome Isolation Kit), which was further identified by Western blotting analysis (CD63/TSG101), transmission electron microscope analysis (TEM) and nanoparticle tracking analysis (NTA). Exosomes were next solubilized for Exo-PD-L1 detection by enzyme-linked immuno-sorbent assay (ELISA). PD-L1 expression in matched tissue were assessed by PD-L1 immunohistochemistry (IHC) (clone 28-8) assay. Tumor proportion score (TPS) ≥ 1% was deemed as “positive” in this study and TPS < 1% was deemed as “negative”. Written informed consent were obtained before acquisition of all data and biological sample. Data were analyzed using SPSS 20.0 and Graphpad Prism 5 software. Chi square test was conducted to estimate the correlation between Exo-PD-L1 levels, sPD-L1 levels, PD-L1 IHC profiles and clinicopathological features. For all analysis, a two-sided *p *< 0.05 was considered significant statistically.

**Results:**

Exo-PD-L1 levels were higher in NSCLC patients with advanced tumor stage, larger tumor size (> 2.5 cm) (*p *< 0.001), positive lymph node status (*p *< 0.05) and distant metastasis (*p *< 0.05). In contrast, sPD-L1 levels were not different between NSCLC patients and healthy donors, it was not correlated with any clinicopathologic features except for tumor size (> 2.5 cm) (*p *< 0.05). In addition, Exo-PD-L1 levels showed slight correlation with sPD-L1 levels (Spearman’s correlation at r = 0.3, *p *= 0.0027) while no correlation with PD-L1 IHC profiles was detected.

**Conclusions:**

In conclusion, Exo-PD-L1, but not sPD-L1, was correlated with NSCLC disease progression, including tumor size, lymph node status, metastasis and TNM stage. However, Exo-PD-L1 was not associated with PD-L1 IHC status.

## Background

Programmed cell-death ligand 1(PD-L1) is an immunosuppressive molecule mainly expressed on the surface of tumor cells [1], which inhibits anti-tumor function of T cells by activating programmed cell-death protein 1 (PD-1)/PD-L1 signaling pathway in the tumor microenvironment (TME) and causes immune escape of tumor cells [2, 3]. Immunotherapy, principally represented by PD-1/PD-L1 inhibitors, has improved the clinical outcome of advanced non-small cell lung cancer (NSCLC) [4–6].

In some kinds of tumor, expression of PD-L1 on tumor cells is associated with clinical response. The PD-L1 immunohistochemistry (IHC) staining is routinely tested as a predictor for anti-PD-1/PD-L1 immune therapy [7]. Compared to that with negative/weak PD-L1 expression, the response rate of high PD-L1 expression group rose from 8 to 30% [8]. Nevertheless, many NSCLC patients with PD-L1 IHC staining positive did not benefit from the immunotherapy. The reasons for this disappointing therapeutic response are unclear, but it is likely that the comprehensive mechanisms of PD-L1-driven pathway in TME, including significance of circulating PD-L1 are not fully understood.

Exosomes are small membrane-bound vesicles with a diameter of 50–150 nm, which protect bioactive molecules including nucleic acids and proteins from degradation in body fluids [9, 10]. These small vesicles are considered to be novel cross-talk circuits established by tumor cells and tumor microenvironment [11–13], and several studies have elucidated that exosomes even represent an immune-inhibitor mechanism in TME [9, 14] as well as participate in tumor progression [15]. However, whether PD-L1 protein is carried by serum-derived exosomes of NSCLC patients and whether Exo-PD-L1 takes part in tumor progression are still unknown.

In this study, we aimed to explore the clinical significance of PD-L1 status both in serum-derived exosomes (Exo-PD-L1) and soluble PD-L1 (sPD-L1) in NSCLC patients. We further explore the relationship among Exo-PD-L1 levels, sPD-L1 levels and PD-L1 IHC staining.

## Materials and methods

### Patient selection

The study enrolled 85 consecutive patients who were administrated from May 2017 to September 2017 at the Departments of Respiratory Medicine and Cardiothoracic Surgery of Jinling Hospital, Nanjing, China. Patients were primarily diagnosed as NSCLC, and had never received any therapies before, including chemotherapy, radiotherapy, targeted therapy or surgical resection. 27 healthy donors were collected from the physical examination center of Jinling Hospital. The lung tumor histology was categorized according to the 2015 World Health Organization (WHO) classification system. This study was approved by the local ethics committee of Jinling hospital. The patients were informed of necessary information concerning this study and signed written informed consent.

### Extraction of exosomes from serum

Overall, 4 ml of blood samples were collected into plain tubes, allowed to clot at 37 °C for 20 min and then were centrifuged at 2000×*g* for 10 min to collect serum. Clear serum was further centrifuged at 10,000×*g* for 30 min to remove cells and debris. The suspension was filtered through 0.22-µm filters (Millipore, Billicera, MA, USA) and stored at − 80 °C until analysis.

The exosomes were isolated according to manufacturer protocol. Briefly, after being thawed in a 25 °C water bath, the samples were added 0.2 volume of Total Exosome Isolation (from serum, Thermo, California USA) reagent. The mixture of serum and reagent were blended gently, incubated at 4 °C for 30 min and centrifuged at 10,000×*g* for 10 min. Finally the exosome pellet was precipitated at the bottom after discarding the supernatant.

### Transmission electron microscopy (TEM) of exosomes uptake

Isolated exosomes were resuspended in PBS. The suspension was placed on a chloroform-coated copper grid with 0.125% Formvar and negatively stained with uranyl acetate. Images were observed under a JEOL 1200EX TEMSCAN electron microscope.

### Nanoparticle tracking analysis

Isolated exosomes were diluted uniformly in PBS solution and were further measured by a NanoSight NS300 Instrument (NanoSight Ltd, Amesbury, United Kingdom) with Nanoparticle Tracking Analysis (NTA) software. Approximately 3 × 10^8^ particles/ml sample were conducted to assess the size distribution and concentration.

### Western blotting analysis

The procedures were conducted as previous described [16]. The isolated exosome pellet was lysed using a lysis buffer which contains the protein extraction reagent RIPA (Beyotime, Nantong, China), PMSF (Roche, Basel, Switzerland) and a protease inhibitor cocktail (Roche, Basel, Switzerland). BCA protein Assay Kit (Thermo Scientific, Rockford, USA) was employed to quantify the total protein concentration. Approximately 30 μg of total protein was electrophoresed on a 10% sodium dodecyl sulfate–polyacrylamide gel and electro-transferred to a PVDF membrane (Millipore). The membrane was then blocked with 5% skim milk for 2 h, immunoblotted with anti-PD-L1 (13684, CST, Danvers, MA, USA), anti-CD63 (ab 193349, Abcam, Cambridge, UK), anti-TSG101 (ab125011, Abcam, Cambridge, UK) and anti-β-actin (Abcam, Cambridge, UK) primary antibody, and incubated with the secondary antibody for 60 min.

### ELISA procedures

Exosomes pellets isolated from 100 μl serum were resuspended using cell extraction buffer (1×), A Human PD-L1 (clone 28-8) ELISA Kit (ab214565, Abcam Cambridge, UK) was used for quantitation of the Exo-PD-L1 and sPDL1 concentration based on the recommendatory procedures. The total protein of exosomes was tested by BCA protein Assay Kit (Thermo Scientific, Rockford, USA). All data were normalized to 1 ml serum.

### Immunohistochemistry

The paraffin-embedded tumor samples were sliced and subjected to immunohistochemical staining for PD-L1 in the Autostainer Link 48 according to protocol. Deparaffinization, rehydration and antigen retrieval were performed according to the PT Link User Guide. Ensuring slides remain wet with buffer while loading and prior to initiating run. The staining process performed by applying anti-PD-L1 antibody (1:400, 28-8 clone, ab205921, Abcam, Cambridge, UK) for 60 min at R.T. and slices were then incubated with The EnVision FLEX Hematoxylin (Link) (Code K8008) for 7 min and DAB (3,3′-diaminobenzidine) were used to detect primary antibody. Slides were incubated with hematoxylin for 3 min after washing and finally dehydrated and coverslipped.

### PD-L1 expression quantification

All areas in each tissue section were observed for proper estimate of the expression of PD-L1. PD-L1 scoring based on the standards for clone 28-8 IHC assay defined as the percentage of tumor cells exhibiting positive membrane staining at any intensity [17]. TPS ≥ 1% was considered “positive” in this study, otherwise TPS < 1% “negative”. The slices for PD-L1 staining were evaluated separately by two lung pathologists, when discrepancy came, the slices were evaluated by another pathologist to meet accordance.

### Statistical analysis

Data were expressed as mean ± SD. Mann–Whitney *U* test was conducted for comparison between groups. Cohen’s kappa coefficient were used to assess the agreement between the evaluations of IHC. Chi square test was conducted to estimate the correlation between Exo-PD-L1 concentration, sPDL1, PD-L1 IHC expression and clinicopathological features. Statistical analysis was performed using SPSS software system (vision 22.0, SPSS, Inc., Chicago, IL) and GraphPad Prism 5 software. For all analysis, a two-sided *p *< 0.05 was considered significant statistically.

## Results

### Clinicopathological features of the study cohort

Lung tissue samples of 85 patients were obtained from diagnostic procedures, and patient characteristics were summarized (Table 1). Adenocarcinoma and squamous carcinoma subtypes accounted for 85.9% and 14.1%, respectively. The information of patients was extracted from the database. Sixty-five (76.5%) of the 85 patients were stage I–IIIA NSCLC, and 20 (23.5%) were stage IIIB–IV NSCLC. In addition, most patients presented with tumor size ≤ 2.5 cm (50.6%), a negative nodal status (62.3%) and negative distant metastasis (85.9%).

**Table 1:** Clinicopathologic parameters of involved NSCLC patients

Characteristics	N (%)
Age (years)	
≤ 60	38 (44.71)
> 60	47 (55.29)
Gender	
Male	46 (54.12)
Female	39 (45.88)
Smoking status	
Smoker	26 (30.59)
Non-smoker	59 (69.41)
Histology	
Squamous carcinoma	12 (14.12)
Adenocarcinoma	73 (85.88)
TNM stage	
I–IIIA	65 (76.47)
IIIB–IV	20 (23.53)
Tumor size (cm)	
≤ 2.5	43 (50.59)
> 2.5	42 (49.41)
Lymph node status	
N0	53 (62.35)
N1–3	32 (37.65)
Distant metastasis	
M0	73 (85.88)
M1	12 (14.12)

### Characterization of exosomes extracted from serum

Exosomes were derived successfully from serum of all patients and healthy volunteers. We chose precipitation method because of limited volume of serum even though ultracentrifugation-isolated exosomes show higher purity. We then verified the isolated exosomes in terms of size, morphology and specific markers.

**Fig. 1:**
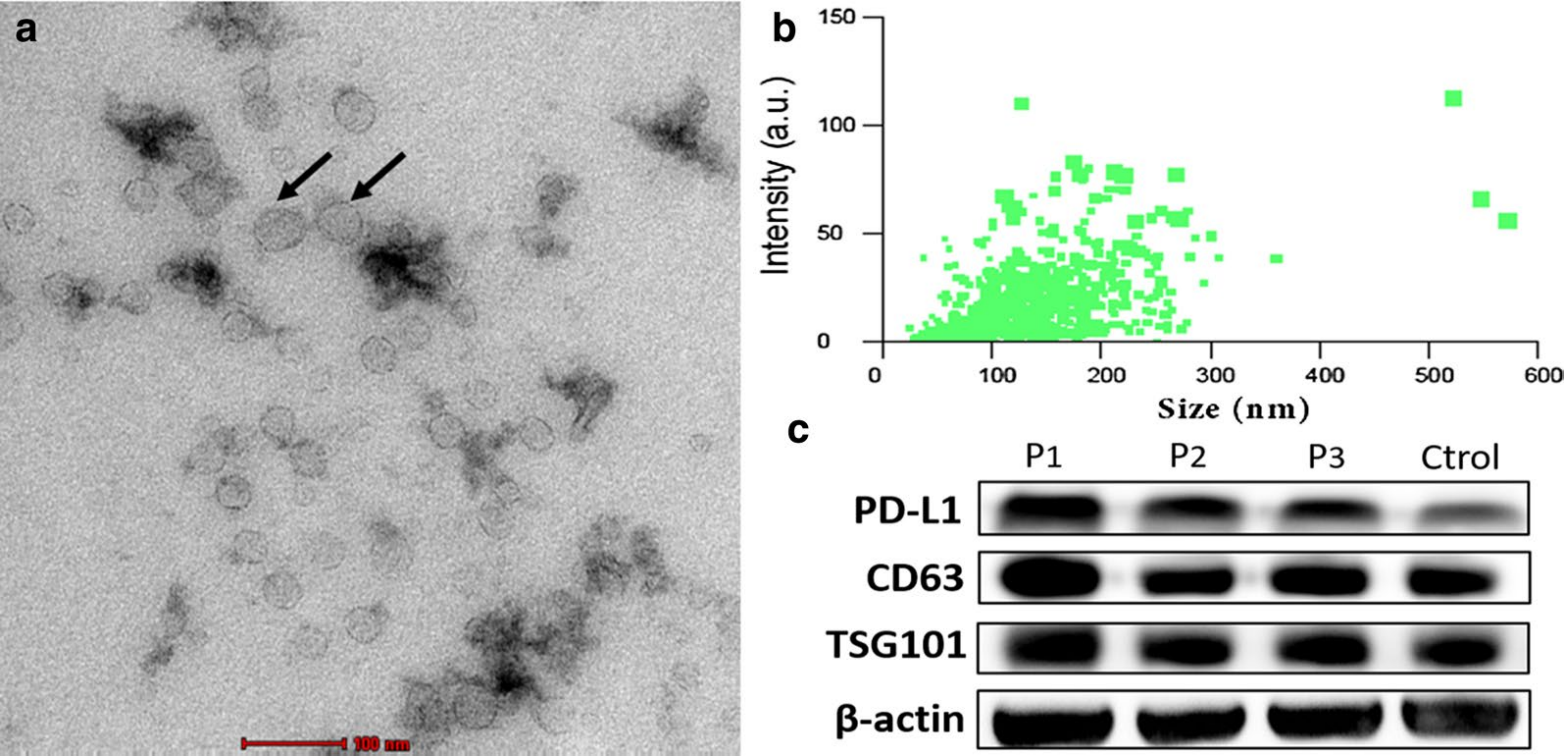
Characterization of serum-derived exosomes of NSCLC. **a**. Exosomes (black arrows) isolated from NSCLC patients were observed under electron microscopy with 50–150 nm in diameter (bar = 100 nm). **b**. Size distribution of exosomes measured by NTA (mean value 121.4 nm). **c** Exosomes-enriched protein CD63/TSG101 and protein PD-L1 were analyzed by western blotting among NSCLC patients and healthy controls

Morphologically, exosomes were spherical, membrane-bound vesicles as described [9] (Fig. 1a). Nanoparticle Tracking Analysis showed that the mode size of exosomes was 121.4 nm (Fig. 1b) and western blotting analysis detected rich expression of exosomal specific markers (CD63 and TSG101) as well as PD-L1 protein in the samples (Fig. 1c). In addition, CD91 as one of lung adenocarcinoma specific antigen expressed on exosomes was also detected (Additional file 1: Figure S1). In summary, this isolation method was eligible for the following experiments.

### The PD-L1 expression in FFPE tissue sections based on the 28-8 –IHC assay

**Fig. 2:**
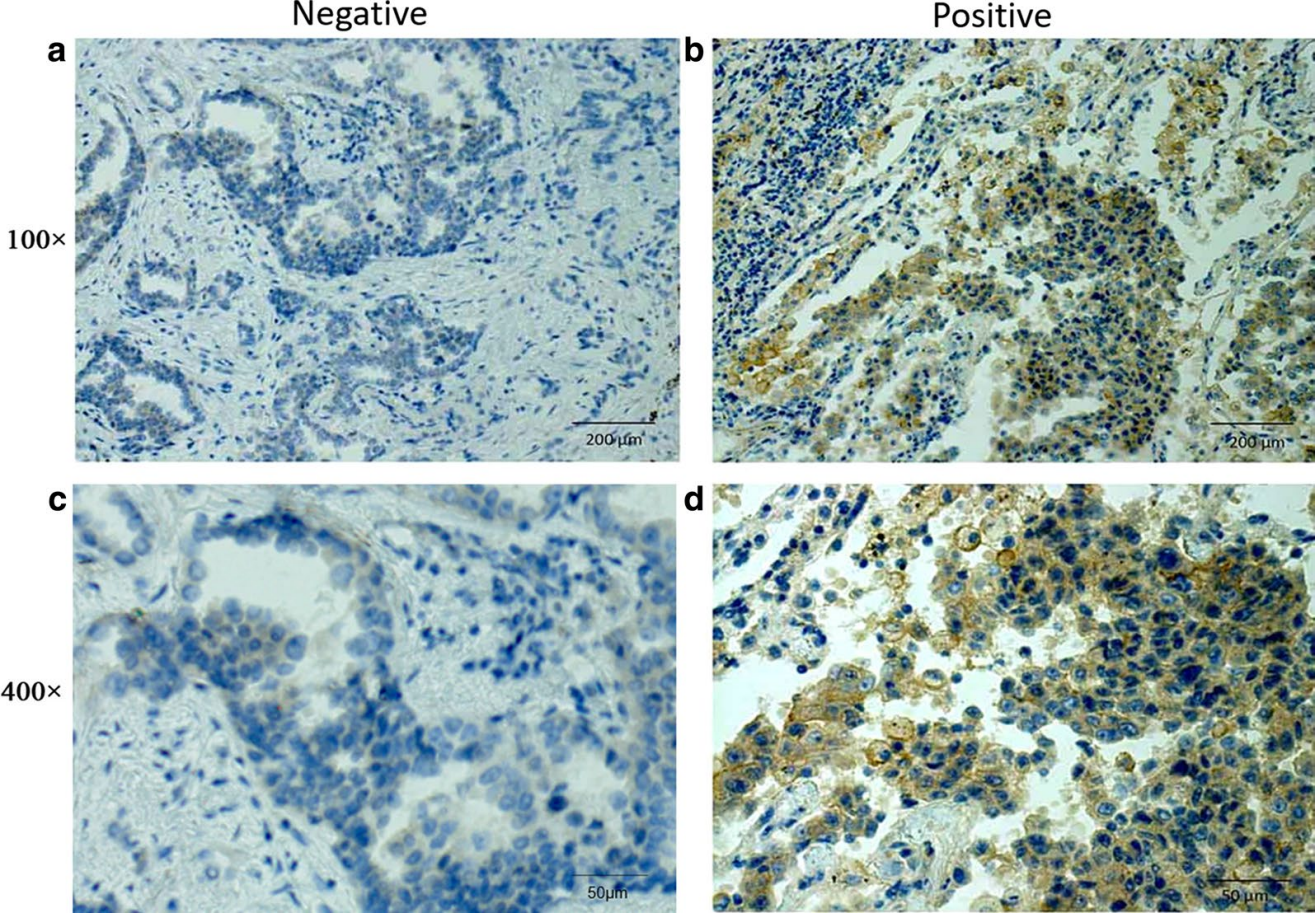
Representative PD-L1 IHC micrographs of negative and positive tumor cells (TCs) staining. **a**, **b** PD-L1-negativity and PD-L1-positivity in TCs (100×). **c**, **d** PD-L1-negativity and PD-L1-positivity in TCs (400×)

PD-L1 status was considered “positive” if TPS ≥ 1% (at any density on the TC membrane) based on the 28-8-IHC assay scoring criteria. A total of 85 samples were adopted for estimation of PD-L1 expression by two pathologists. The Cohen’s Kappa coefficient analysis was conducted and a Kappa coefficient of 0.761 (*p *< 0.001) showed fair agreement of two pathologist’s judgement. Discrepancy occurred among 9 of 85 slices and these samples were evaluated by a third pathologist. In general, the PD-L1-positive specimens accounted for 23(27.0%) and negative 62(73.0%) (Additional file 2: Table S1). The 28-8 IHC staining of slices was observed under microscopy. The images respectively represented PD-L1-negative and PD-L1-positive staining at 100 × magnification (Fig. 2a, b), PD-L1-negative and PD-L1-positive staining at 400× magnification (Fig. 2c, d), respectively. Furthermore, Image-Pro Plus 6.0 software was applied on PD-L1 IHC quantitative evaluation, three pictures respectively showed with staining density of 0% (Negative), 6.4% (Positive1) and 66.3% (Positive2) (Addtional file 3: Figure S2).

### Concentration of Exo-PD-L1 and sPDL1 in serum of NSCLC

**Fig. 3:**
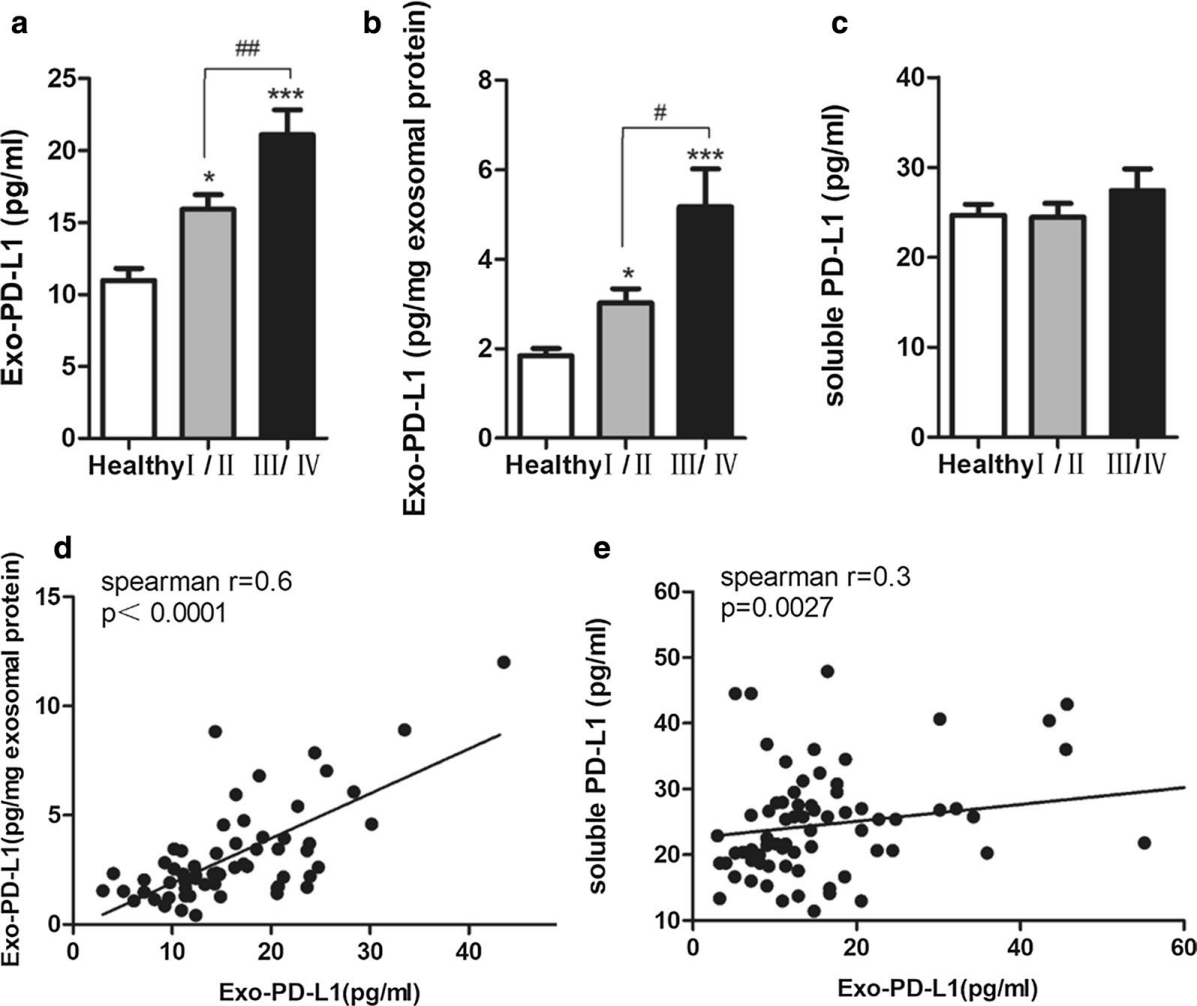
Correlation between Exo-PD-L1 and sPDL1 profiles. **a**, **b** Quantitative analysis of Exo-PD-L1 levels (pg/ml serum) (**a**) and relative Exo-PD-L1 levels (pg/mg exosomal protein) (**b**) among healthy individuals (n = 27), stage I–II (n = 57) and III/IV (n = 28) NSCLC patients; *p < 0.05; ***p < 0.001; ^#^*p *< 0.05; ^##^*p *< 0.01. **c** sPDL1 levels were not statistically different in NSCLC patients from healthy donors. **d** Correlation between Exo-PD-L1 and relative Exo-PD-L1 levels; Spearman’s correlation at r = 0.6, *p *< 0.0001. **e** Correlation between Exo-PD-L1 and sPD-L1 levels; Spearman’s correlation at r = 0.3, *p *= 0.0027

The profile of Exo-PD-L1 was determined by ELISA and the results showed that Exo-PD-L1 levels of I-II (15.90 ± 6.45 pg/ml serum) and III/IV NSCLC patients (21.10 ± 11.63 pg/ml serum) were considerably higher than that of healthy controls (15.91 ± 6.45 pg/ml serum) (p < 0.05 and p < 0.001, respectively) (Fig. 3a). The concentration was further normalized to per milligram exosomal protein, namely the ratio of Exo-PD-L1 to exosomal protein, which showed association with corresponding Exo-PD-L1 concentration (Fig. 3d). Similarly, significant difference was found in Exo-PD-L1 between NSCLC patients and healthy controls (1.84 ± 0.72 pg/mg exosomal protein) with a median concentration of 3.02 ± 1.67 pg/mg exosomal protein for I-II NSCLC and 5.17 ± 3.16 pg/mg exosomal protein for III/IV NSCLC) (Fig. 3b).

We also explored the levels of sPDL1 by ELISA in 48 of 85 NSCLC patients and 27 healthy controls. However, no significant difference was detected in sPDL1 profile between NSCLC patients and healthy controls (Fig. 3c). In addition, we found an association between Exo-PD-L1 and sPDL1 concentration with an R value of 0.3 (*p *= 0.0027) (Fig. 3e).

### Correlation analysis of Exo-PD-L1, sPDL1, PD-L1 IHC status and clinicopathological parameters of NSCLC

**Fig. 4:**
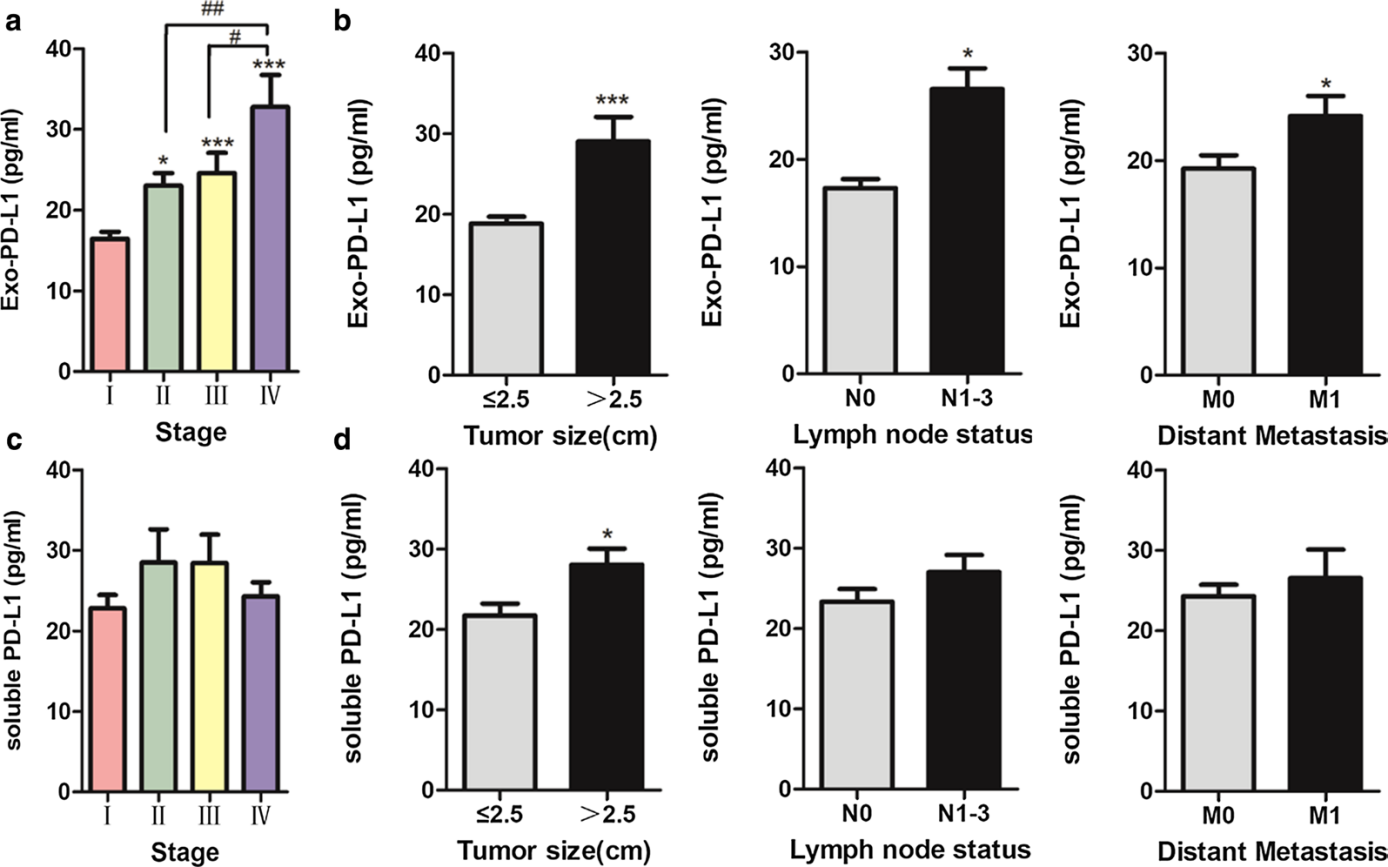
Exo-PD-L1 and sPDL1 levels in serum of NSCLC patients. **a**, **b** Exo-PD-L1 levels were higher in NSCLC patients with advanced stage (**a**), larger tumor size (> 2.5 cm), positive lymph node status (N1–N3) and distant metastasis (M1) (**b**); *p < 0.05; ***p < 0.001; ^#^*p *< 0.05; ^##^*p *< 0.01. **c**, **d** sPDL1 levels were not significantly different in NSCLC patients stratified by stage (**c**), lymph node status and metastasis except for tumor size (> 2.5 cm) (**d**); *p < 0.05

Correlation between Exo-PD-L1, sPDL1 concentration and several clinicopathological characteristics (age, gender, smoking status, histologic subtype, tumor size, lymph node status, distant metastasis and TNM stage) were investigated. The results were summarized in Table 2. Generally, higher Exo-PD-L1 was associated with advanced TNM stage, larger tumor size, positive lymph node status and distant metastasis (Fig. 4a, b). Specifically, sPDL1 was correlated with tumor size. Stage II/III/IV tumor had significantly higher sPDL1 levels than stage I while ANOVA test showed that sPDL1 expression was not different between each group (Fig. 4c, d). No other clinical variables were significantly associated with Exo-PD-L1 profiles (Additional file 4: Figure S3).

**Table 2:** Correlations between Exo-PD-L1, soluble PD-L1 and clinicopathological features

Characteristics	N	EXO-PD-L1			N	Soluble PD-L1		
		Low N (%)	High N (%)	*p*-value		Low N (%)	High N (%)	*p*-value
Age (years)				0.528				0.083
≤ 60	38	20 (23.53)	18 (21.18)		24	15 (31.25)	9 (18.75)	
> 60	47	22 (25.88)	25 (29.41)		24	9 (18.75)	15 (31.25)	
Gender				0.063				0.233
Male	46	27 (32.94)	19 (21.18)		30	17 (35.42)	13 (27.08)	
Female	39	15 (16.47)	24 (9.41)		18	7 (14.58)	11 (22.92)	
Smoking status				0.138				0.143
Smoker	26	16 (18.82)	10 (11.77)		14	8 (16.67)	6 (12.50)	
Non-smoker	59	26 (30.59)	33 (38.82)		34	16 (33.33)	18 (37.50)	
Histology				0.505				0.348
Squamous carcinoma	12	7 (8.24)	5 (5.88)		5	1 (2.08)	4 (8.33)	
Adenocarcinoma	73	35 (41.18)	38 (44.70)		43	23 (47.92)	20 (41.67)	
TNM stage				0.012*				0.140
I	47	29 (34.12)	18 (21.18)		29	17(37.50)	12 (22.92)	
II/III/IV	38	13 (15.29)	25 (29.41)		19	7 (12.50)	12 (27.08)	
Tumor size (cm)				0.003**				0.004**
≤ 2.5	43	28 (32.94)	15 (17.65)		26	18 (37.50)	8 (16.67)	
> 2.5	42	14 (16.47)	28 (32.94)		22	6 (12.50)	16 (33.33)	
Lymph node status				0.031*				0.131
N0	53	31 (36.47)	22 (25.88)		31	18 (37.50)	13 (27.08)	
N1–3	32	11 (12.94)	21 (24.71)		17	6 (12.50)	11 (22.92)	
Distant metastasis				0.026*				0.220
M0	73	40 (47.06)	33 (38.82)		41	22 (45.83)	19 (39.58)	
M1	12	2 (2.35)	10 (11.77)		7	2 (4.17)	5 (10.42)	

* p < 0.05, ** p < 0.01

We further evaluated the correlation between PD-L1 expression and the clinicopathological features of 85 NSCLC patients. The results showed that PD-L1 expression was significantly correlated with smoking status (*p *= 0.037) and histology subtype (*p *= 0.012). However, we found no significant association between age, gender, tumor size, lymph node metastasis, TNM stage and PD-L1 IHC status (Additional file 5: Table S2).

### Correlation analysis of Exo-PD-L1, sPDL1 and PD-L1 IHC staining

**Fig. 5:**
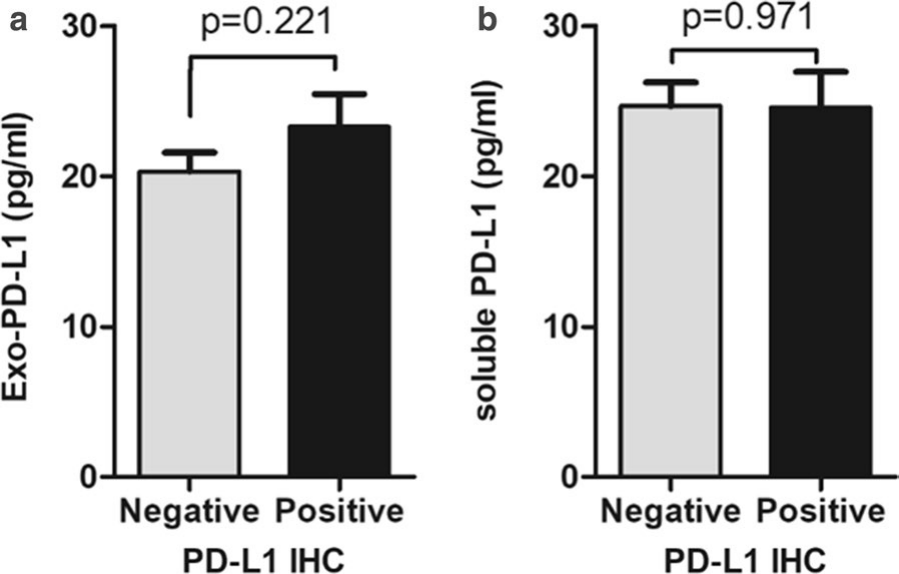
Comparison of Exo-PD-L1 and sPDL1 levels between PD-L1 IHC staining positivity and negativity. **a**, **b** Exo-PD-L1 (**a**) and sPDL1 (**b**) levels in the PD-L1 IHC positive group (n = 23) and negative group (n = 62) of NSCLC patients

We next investigated the correlation between Exo-PD-L1, sPDL1 concentration and PD-L1 IHC status in matched samples based on 28-8-IHC assay/28-8 ELISA kit. No significant difference was observed in the expression of Exo-PD-L1 or sPDL1 between PD-L1 IHC negativity and positivity groups (Fig. 5a, b).

## Discussion

In summary, we explored the clinical value of Exo-PD-L1, sPDL1 and PD-L1 profile. Our data showed that NSCLC patients, especially those with advanced stage, obtained higher levels of Exo-PD-L1 than healthy controls. Furthermore, higher Exo-PD-L1 content was associated with larger tumor size, positive lymph node status, distant metastasis and advanced TNM stage in NSCLC patients. However, sPDL1 did not differ among each stage of NSCLC patients and healthy controls. Our data also showed that Exo-PD-L1 was slightly correlated with sPDL1 (Additional file 4: Figure S2).

Exosomes are extracellular vesicles found in blood, urine and other body fluid [18]. They are secreted excessively by tumor cells under oxidative stress [19] and participate in cross-talk with the microenvironment of cancer [20]. As a key communicator, exosomes are rich of bioactive molecules [21] including immune checkpoint molecules. Cancer cells were confirmed to release exosomes carrying PD-1, PD-L1 or cytotoxic lymphocyte antigen 4 (CTLA-4) [14, 15, 22–24]. Our data agreed with previous studies and confirmed that PD-L1 was also expressed in circulating exosomes of NSCLC patients (Additional file 5: Figure S3).

Actually, PD-L1 in circulating exosomes had been proved to have certain clinical potential and regulate biological functions in tumor. In our study, we showed that NSCLC patients had elevated Exo-PD-L1 levels than healthy controls. A significant positive association was found between Exo-PD-L1 expression and clinicopathologic disease indicators including tumor size, lymph node status, distant metastasis and TNM stage. Similarly, several studies [22–24] found PD-L1 levels carried by exosomes correlated with disease progression. Gang Chen et al. [25] also demonstrated that exosomal PD-L1 positively correlated with tumor size and poor prognosis.

In contrast, sPDL1 concentration in NSCLC patients of each stage did not differ from that in healthy donors, which was consistent with the study of Theodoraki et al. [23]. Another study conducted by Zhou et al. [26] clarified that sPDL1 levels were elevated in stage IV melanoma patients than in healthy donors. In our study, sPDL1 did not correlate with any clinicopathologic features except for tumor size. Furthermore, it sPDL1 levels were slightly correlated with Exo-PD-L1 levels. This phenomenon might be explained by the machinery they formed and roles of these two different types of PD-L1 played in the TME.

Endogenous form of PD-L1 was specifically secreted within exosomes [27]. It was released into the lymph node to suppress anti-tumor immunity by inhibiting T cell activity [28, 29]. Namely, Exo-PD-L1 helped to form pre-metastatic tumor microenvironment. Blocking the secretion of Exo-PD-L1 could suppress both growth of the local tumor cells and distant metastasis [27]. While sPDL1 was generated as a splice variant lacking of function [30, 31]. The mechanisms by which sPDL1 was produced and the function of sPDL1 remain poorly understood. It might be the discrepancy in their biogenesis resulted in weak relationship between Exo-PD-L1 and sPDL1.

Our study for the first time compared the correlation between tumor PD-L1 IHC profiles and levels of Exo-PD-L1 and levels of sPDL1, respectively. We confirmed that neither Exo-PD-L1 nor sPDL1 was differently expressed between PD-L1 IHC positive and negative group. One explanation might be that the expression of PD-L1 in tumor sample is heterogeneous [32, 33] and exosomes could be secreted by several kinds of cells other than tumor cells [34]. Therefore, there was no correlation between Exo-PD-L1 and PD-L1 IHC profiles. Several papers have reported that circulating Exo-PD-L1 played a vital role in anti-PD-1/PD-L1 blockade therapy. Poggio et al. [27] discovered that Exo-PD-L1 appeared to be resistant to anti-PD-L1 therapy. In the meantime, the inhibition of Exo-PD-L1 could help maintain a long-lasting anti-tumor immunity. One study [35] explored the relationship of the exosomal PD-L1 mRNA expression with response to anti-PD-1 therapy in both melanoma (n = 18) and NSCLC (n = 8). They demonstrated that exosomal PD-L1 mRNA levels changed dynamically during treatment with nivolumab and pembrolizumab. They further emphasized that Exo-PD-L1 should be considered in predicting outcome of anti-PD-1 therapy. This might explained why PD-L1 IHC profiles of tumor is not an ideal biomarker to select candidates for anti-PD-1/PD-L1 immune therapy [36, 37].

Our study also had certain limitations. (i) The majority of the patients included in this study was early-stage NSCLC patients, thereby introducing certain bias for correlation analysis. (ii) None of the patients included in this study had previous PD-1 or PD-L1 immune therapy sections. Therefore, we could not investigate the influence Exo-PD-L1 had on anti-PD-1/PD-L1 blockade therapy.

In conclusion, our study explored the clinical significance of circulating forms of PD-L1, including Exo-PD-L1 and soluble PD-L1 in NSCLC. We found that Exo-PD-L1 but not sPD-L1, was correlated with NSCLC disease progression. In addition, we discovered for the first time Exo-PD-L1 concentration was not statistically correlated with PD-L1 IHC status. Further large-sampled studies exploring the relationship between Exo-PD-L1 and the outcome of pre/post anti-PD-1/PD-L1 blockade therapy are needed to validate the clinical potential of Exo-PD-L1.

## Conclusions

In summary, Exo-PD-L1, instead of sPD-L1, was higher in NSCLC patients with advanced TNM tumor stage, larger tumor size (> 2.5 cm), positive lymph node status and distant metastasis. However, Exo-PD-L1 and sPD-L1 was not correlated with PD-L1 IHC status.

## Supplementary information

**Additional file 1: Figure S1.** CD91 were analyzed by western blot among Lung adenocarcinoma and healthy control.

**Additional file 2: Table S1.** PD-L1 IHC scoring according to two pathologists’ judgement.

**Additional file 3: Figure S2.** PD-L1 IHC quantitative evaluation was tested by Image-Pro Plus 6.0 software.

**Additional file 4: Figure S3.** Exo-PD-L1 (a) and sPDL1 (b) levels in serum of NSCLC patients.

**Additional file 5: Table S2.** Correlations between PD-L1 IHC profiles and clinicopathological features.

## Data Availability

We declared that materials described in the manuscript, including relevant data, will be freely available to any scientist wishing to use them.

## Abbreviations

CTLA-4: cytotoxic lymphocyte antigen 4
ELISA: enzyme-linked immuno-sorbent assay
Exo-PD-L1: exosomal PD-L1
IHC: immunohistochemistry
NSCLC: non-small cell lung cancer
NTA: nanoparticle tracking analysis
PD-1: programmed cell-death protein 1
PD-L1: programmed cell-death ligand 1
sPD-L1: soluble PD-L1
TC: tumor cell
TEM: transmission electron microscope analysis
TME: tumor microenvironment
TPS: tumor proportion score
WHO: world health organization

## References

[1] Wang X, Teng F, Kong L, Yu J (2016). PD-L1 expression in human cancers and its association with clinical outcomes. OncoTargets Therapy..

[2] Mittal D, Gubin MM, Schreiber RD, Smyth MJ (2014). New insights into cancer immunoediting and its three component phases–elimination, equilibrium and escape. Curr Opin Immunol.

[3] Ichikawa M, Chen L (2005). Role of B7-H1 and B7-H4 molecules in down-regulating effector phase of T-cell immunity: novel cancer escaping mechanisms. Front Biosci..

[4] Herbst RS, Baas P, Kim DW, Felip E, Perez-Gracia JL, Han JY (2016). Pembrolizumab versus docetaxel for previously treated, PD-L1-positive, advanced non-small-cell lung cancer (KEYNOTE-010): a randomised controlled trial. Lancet..

[5] Brahmer JR, Rodriguez-Abreu D, Robinson AG, Hui R, Csoszi T, Fulop A (2017). Health-related quality-of-life results for pembrolizumab versus chemotherapy in advanced, PD-L1-positive NSCLC (KEYNOTE-024): a multicentre, international, randomised, open-label phase 3 trial. Lancet Oncol.

[6] De Lima Lopes G, Wu YL, Sadowski S, Zhang J, Rangwala R, Kush D (2016). P2.43 Pembrolizumab vs platinum-based chemotherapy for PD-L1 + NSCLC: phase 3, randomized, open-label KEYNOTE-042 (NCT02220894): track: immunotherapy. J Thorac Oncol..

[7] Ilie M, Long-Mira E, Bence C, Butori C, Lassalle S, Bouhlel L (2016). Comparative study of the PD-L1 status between surgically resected specimens and matched biopsies of NSCLC patients reveal major discordances: a potential issue for anti-PD-L1 therapeutic strategies. Ann Oncol..

[8] Mezquita L, Auclin E, Ferrara R, Charrier M, Remon J, Planchard D (2018). Association of the lung immune prognostic index with immune checkpoint inhibitor outcomes in patients with advanced non-small cell lung cancer. JAMA Oncol..

[9] Whiteside TL (2016). Exosomes and tumor-mediated immune suppression. J Clin Investig..

[10] Guay C, Regazzi R (2017). Exosomes as new players in metabolic organ cross-talk. Diabetes Obes Metab.

[11] Li Y, Zheng Q, Bao C, Li S, Guo W, Zhao J (2015). Circular RNA is enriched and stable in exosomes: a promising biomarker for cancer diagnosis. Cell Res.

[12] Melo SA, Luecke LB, Kahlert C, Fernandez AF, Gammon ST, Kaye J (2015). Glypican-1 identifies cancer exosomes and detects early pancreatic cancer. Nature.

[13] Tang MK, Wong AS (2015). Exosomes: emerging biomarkers and targets for ovarian cancer. Cancer Lett.

[14] Hong CS, Funk S, Muller L, Boyiadzis M, Whiteside TL (2016). Isolation of biologically active and morphologically intact exosomes from plasma of patients with cancer. J Extracell Vesicles..

[15] Ludwig S, Floros T, Theodoraki MN, Hong CS, Jackson EK, Lang S (2017). Suppression of lymphocyte functions by plasma exosomes correlates with disease activity in patients with head and neck cancer. Clin Cancer Res..

[16] Gao J, Qiu X, Li X, Fan H, Zhang F, Lv T (2018). Expression profiles and clinical value of plasma exosomal Tim-3 and Galectin-9 in non-small cell lung cancer. Biochem Biophys Res Commun.

[17] Buttner R, Gosney JR, Skov BG, Adam J, Motoi N, Bloom KJ (2017). Programmed death-ligand 1 immunohistochemistry testing: a review of analytical assays and clinical implementation in non-small-cell lung cancer. J Clin Oncol.

[18] Boukouris S, Mathivanan S (2015). Exosomes in bodily fluids are a highly stable resource of disease biomarkers. Proteomics Clin Appl..

[19] King HW, Michael MZ, Gleadle JM (2012). Hypoxic enhancement of exosome release by breast cancer cells. BMC Cancer..

[20] Haderk F, Schulz R, Iskar M, Cid LL, Worst T, Willmund KV (2017). Tumor-derived exosomes modulate PD-L1 expression in monocytes. Sci Immunol..

[21] Becker A, Thakur BK, Weiss JM, Kim HS, Peinado H, Lyden D (2016). Extracellular vesicles in cancer: cell-to-cell mediators of metastasis. Cancer Cell.

[22] Ricklefs FL, Alayo Q, Krenzlin H, Mahmoud AB, Speranza MC, Nakashima H (2018). Immune evasion mediated by PD-L1 on glioblastoma-derived extracellular vesicles. Sci Adv..

[23] Theodoraki MN, Yerneni SS, Hoffmann TK, Gooding WE, Whiteside TL (2018). Clinical significance of PD-L1(+) exosomes in plasma of head and neck cancer patients. Clin Cancer Res..

[24] Yang Y, Li CW, Chan LC, Wei Y, Hsu JM, Xia W (2018). Exosomal PD-L1 harbors active defense function to suppress T cell killing of breast cancer cells and promote tumor growth. Cell Res.

[25] Chen G, Huang AC, Zhang W, Zhang G, Wu M, Xu W (2018). Exosomal PD-L1 contributes to immunosuppression and is associated with anti-PD-1 response. Nature.

[26] Zhou J, Mahoney KM, Giobbie-Hurder A, Zhao F, Lee S, Liao X (2017). Soluble PD-L1 as a biomarker in malignant melanoma treated with checkpoint blockade. Cancer Immunol Res.

[27] Poggio M, Hu T, Pai CC, Chu B, Belair CD, Chang A (2019). Suppression of exosomal PD-L1 induces systemic anti-tumor immunity and memory. Cell.

[28] Guo Y, Ji X, Liu J, Fan D, Zhou Q, Chen C (2019). Effects of exosomes on pre-metastatic niche formation in tumors. Mol Cancer..

[29] Muller L, Simms P, Hong CS, Nishimura MI, Jackson EK, Watkins SC (2017). Human tumor-derived exosomes (TEX) regulate Treg functions via cell surface signaling rather than uptake mechanisms. Oncoimmunology..

[30] van der Voort R, Verweij V, de Witte TM, Lasonder E, Adema GJ, Dolstra H (2010). An alternatively spliced CXCL16 isoform expressed by dendritic cells is a secreted chemoattractant for CXCR31 + cells. J Leukoc Biol.

[31] Venables JP (2004). Aberrant and alternative splicing in cancer. Cancer Res.

[32] Casadevall D, Clave S, Taus A, Hardy-Werbin M, Rocha P, Lorenzo M (2017). Heterogeneity of tumor and immune cell PD-L1 expression and lymphocyte counts in surgical NSCLC samples. Clin Lung Cancer..

[33] Xu H, Lin G, Huang C, Zhu W, Miao Q, Fan X (2017). Assessment of concordance between 22C3 and SP142 immunohistochemistry assays regarding PD-L1 expression in non-small cell lung cancer. Sci Rep..

[34] Ruivo CF, Adem B, Silva M, Melo SA (2017). The biology of cancer exosomes: insights and new perspectives. Cancer Res.

[35] Del Re M, Marconcini R, Pasquini G, Rofi E, Vivaldi C, Bloise F (2018). PD-L1 mRNA expression in plasma-derived exosomes is associated with response to anti-PD-1 antibodies in melanoma and NSCLC. Br J Cancer.

[36] Ribas A, Hamid O, Daud A, Hodi FS, Wolchok JD, Kefford R (2016). Association of pembrolizumab with tumor response and survival among patients with advanced melanoma. JAMA.

[37] Zaretsky JM, Garcia-Diaz A, Shin DS, Escuin-Ordinas H, Hugo W, Hu-Lieskovan S (2016). Mutations associated with acquired resistance to pd-1 blockade in melanoma. N Engl J Med..

