# Supplementary figures and images for: Clinical significance of PD-L1 expression in serum-derived exosomes in NSCLC patients
Source: J Transl Med. 2019 Oct 29;17:355. doi: 10.1186/s12967-019-2101-2 (PMC6820965; doi:10.1186/s12967-019-2101-2)

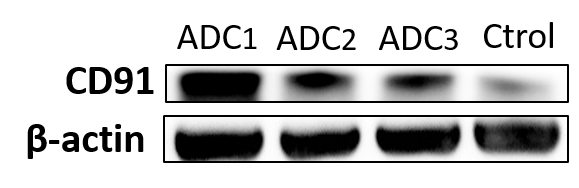

Supplement: Supplementary file 1 — Additional file 1: Figure S1. CD91 were analyzed by western blot among Lung adenocarcinoma and healthy control. [file 12967_2019_2101_MOESM1_ESM.tif]

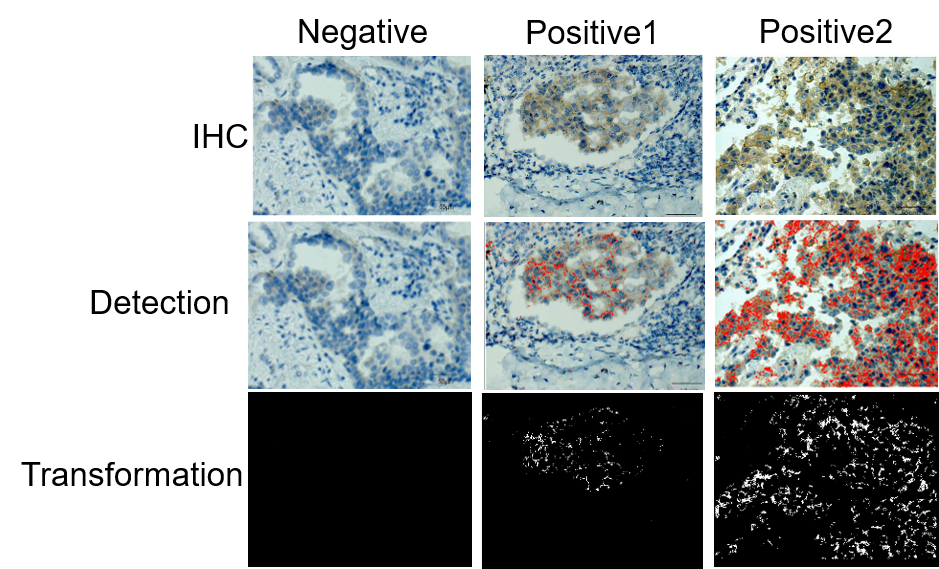

Supplement: Supplementary file 3 — Additional file 3: Figure S2. PD-L1 IHC quantitative evaluation was tested by Image-Pro Plus 6.0 software. [file 12967_2019_2101_MOESM3_ESM.tif]

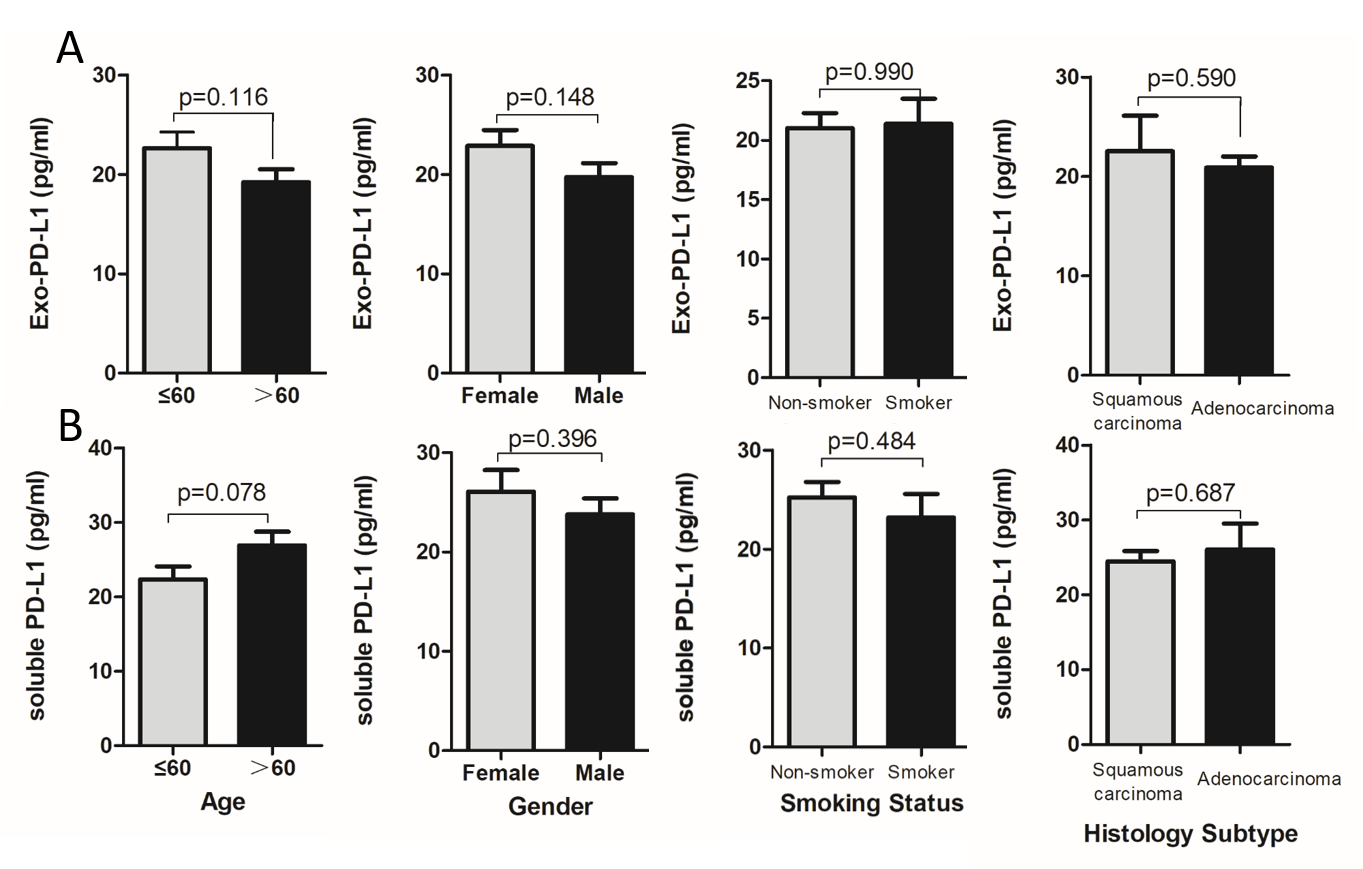

Supplement: Supplementary file 4 — Additional file 4: Figure S3. Exo-PD-L1 (a) and sPDL1 (b) levels in serum of NSCLC patients. [file 12967_2019_2101_MOESM4_ESM.tif]
