# Supplementary material for: Clinical significance of PD-L1 expression in serum-derived exosomes in NSCLC patients
Source: J Transl Med. 2019 Oct 29;17:355. doi: 10.1186/s12967-019-2101-2 (PMC6820965; doi:10.1186/s12967-019-2101-2)
Supplement: Supplementary file 2 — Additional file 2: Table S1. PD-L1 IHC scoring according to two pathologists’ judgement. [file 12967_2019_2101_MOESM2_ESM.docx]

Additional file 2 Table S1. PD-L1 IHC scoring according to two pathologists’ judgement.

|  | Positive | Negative | Total | Kappa value P value |
| --- | --- | --- | --- | --- |
| Positive | 23 | 0 | 23 | 0.761 *P*<0.001 |
| Negative | 9 | 53 | 62 |  |
| Total | 32 | 53 | 85 |  |
