# Supplementary material for: Clinical significance of PD-L1 expression in serum-derived exosomes in NSCLC patients
Source: J Transl Med. 2019 Oct 29;17:355. doi: 10.1186/s12967-019-2101-2 (PMC6820965; doi:10.1186/s12967-019-2101-2)
Supplement: Supplementary file 5 — Additional file 5: Table S2. Correlations between PD-L1 IHC profiles and clinicopathological features. [file 12967_2019_2101_MOESM5_ESM.docx]

Additional file 5 Table S2. Correlations between PD-L1 IHC profiles and clinicopathological features.

| Characteristics | N | PD-L1 IHC Profiles | | |
| --- | --- | --- | --- | --- |
|  |  | Positive  N (%) | Negative  N (%) | *P*-  value |
| Age (years) |  |  |  | 0.402 |
| ≤ 60 | 38 | 9(10.59) | 29(34.12) |  |
| ＞60 | 47 | 15(17.65) | 32(37.64) |  |
| Gender |  |  |  | 0.331 |
| Male | 46 | 15(17.65) | 31(36.47) |  |
| Female | 39 | 9(10.59) | 30(35.29) |  |
| Smoking status |  |  |  | 0.037* |
| Smoker | 25 | 11(12.94) | 14(16.47) |  |
| Non-smoker | 60 | 13(15.29) | 47(55.29) |  |
| Histology |  |  |  | 0.012* |
| Squamous carcinoma | 12 | 7(8.24) | 5(5.88) |  |
| Adenocarcinoma | 73 | 17(20.00) | 56(65.88) |  |
| TNM stage |  |  |  | 0.713 |
| Ⅰ-ⅢA | 65 | 19(22.35) | 46(55.30) |  |
| ⅢB-Ⅳ | 20 | 5(5.88) | 15(16.47) |  |
| Tumor size(cm) |  |  |  | 0.130 |
| ≤ 2.5 | 43 | 9(10.59) | 34(40.00) |  |
| ＞2.5 | 42 | 15(17.65) | 27(31.76) |  |
| Lymph node status |  |  |  | 0.607 |
| N0 | 53 | 16(18.82) | 37(43.53) |  |
| N1-3 | 32 | 8(9.41) | 24(28.24) |  |
| Distant Metastasis |  |  |  | 0.337 |
| M0 | 73 | 22(25.88) | 51(60.00) |  |
| M1 | 12 | 2(2.35) | 10(11.77) |  |
| Type of specimens |  |  |  | 0.536 |
| Surgical resection | 71 | 21(24.71) | 50(58.82) |  |
| Small biopsy | 14 | 3(3.53) | 11(12.94) |  |
| *p<0.05 |  |  |  |  |
